# PRimary care rEsponse to domestic violence and abuse in the COvid-19 panDEmic (PRECODE): protocol of a rapid mixed-methods study in the UK

**DOI:** 10.1186/s12875-021-01447-3

**Published:** 2021-05-12

**Authors:** Eszter Szilassy, Estela Capelas Barbosa, Sharon Dixon, Gene Feder, Chris Griffiths, Medina Johnson, Anna De Simoni, Vari Wileman, Jasmina Panovska-Griffiths, Anna Dowrick

**Affiliations:** 1 Centre for Academic Primary Care, Bristol Medical School, Population Health Sciences, University of Bristol, Canynge Hall, 39 Whatley Road, BS8 2PS Bristol, UK; 2 IRISi, Bristol, UK; 3 Nuffield Department of Primary Care Health Sciences, University of Oxford, Oxford, UK; 4 Donnington Medical Partnership, Oxford, UK; 5 Institute of Population Health Sciences, Barts and The London School of Medicine and Dentistry, Queen Mary University of London, London, UK; 6 Department of Applied Health Research, Institute of Epidemiology and Health Care, University College London, London, UK; 7 Wolfson Centre for Mathematical Biology and The Queen’s College, University of Oxford, Oxford, UK

**Keywords:** COVID-19 pandemic, Primary care, General practice, Domestic violence and abuse, Remote consultation, Referral, Mixed-method protocol, Rapid qualitative analysis, Rapid synthesis, Interrupted-time series analysis

## Abstract

**Background:**

The implementation of lockdowns in the UK during the COVID-19 pandemic resulted in a system switch to remote primary care consulting at the same time as the incidence of domestic violence and abuse (DVA) increased. Lockdown-specific barriers to disclosure of DVA reduced the opportunity for DVA detection and referral.

The PRECODE (PRimary care rEsponse to domestic violence and abuse in the COvid-19 panDEmic) study will comprise quantitative analysis of the impact of the pandemic on referrals from IRIS (Identification and Referral to Improve Safety) trained general practices to DVA agencies in the UK and qualitative analysis of the experiences of clinicians responding to patients affected by DVA and adaptations they have made transitioning to remote DVA training and patient support.

**Methods/Design:**

Using a rapid mixed method design, PRECODE will explore and explain the dynamics of DVA referrals and support before and during the pandemic on a national scale using qualitative data and over four years of referrals time series data.

We will undertake interrupted-time series and non-linear regression analysis, including sensitivity analyses, on time series of referrals to DVA services from routinely collected data to evaluate the impact of the pandemic and associated lockdowns on referrals to the IRIS Programme, and analyse key determinants associated with changes in referrals.

We will also conduct an interview- and observation-based qualitative study to understand the variation, relevance and feasibility of primary care responses to DVA before and during the pandemic and its aftermath.

The triangulation of quantitative and qualitative findings using rapid analysis and synthesis will enable the articulation of multiscale trends in primary care responses to DVA and complex mechanisms by which these responses have changed during the pandemic.

**Discussion:**

Our findings will inform the implementation of remote primary care and DVA service responses as services re-configure. Understanding the adaptation of clinical and service responses to DVA during the pandemic is crucial for the development of evidence-based, effective remote support and referral beyond the pandemic.

**Trial registration:**

PRECODE is an observational epidemiologic study, not an intervention evaluation or trial. We will not be reporting results of an intervention on human participants.

## Background

### Domestic violence and abuse during the COVID-19 pandemic

The global incidence of domestic violence and abuse (DVA) has increased notably during the COVID-19 pandemic in association with strict social distancing measures (lockdowns), widespread anxiety, increased economic precarity, and reduced access to support services [1–7]. While lockdown measures are necessary from a wider public health perspective [8], shutting down or limiting usual routes to support and safety could have a detrimental impact on the health and wellbeing of people affected by DVA and their families [9, 10].

Although DVA prevalence figures largely differ internationally due to variations in measurement techniques [11], even before the pandemic the incidence was high globally [11–13]. In the UK, according to the Office for National Statistics (ONS), 7.3% of women and 3.6% of men nationally have experienced DVA in a year [14] and life-time population prevalence is consistently higher for women seeking health care [15], including primary care [16]. Women experience more repeated and severe abuse, sexual violence, and coercive control [17].

DVA is a violation of human rights that damages health and wellbeing. It requires a multi-sectoral response, including a vital role of health care services in responding to affected patients [18–21]. A major part of this health care response is identification of people who are affected by abuse and enabling access to appropriate support to enhance their safety and improve their health outcomes. In the United Kingdom (UK), that specialist support largely comes from third sector DVA agencies.

### Primary health care response to DVA

There are international [22] and national UK [23] guidelines for the healthcare of women affected by DVA, and a growing recognition of its impact on their health [24, 25]. Over the past decade, in line with guidelines, UK general practice has started to engage with DVA, with training of general practice teams in the identification, support and referral of female patients affected by abuse from a partner/spouse or other adult in the household.

General practice can provide a safe and confidential place for disclosure and enquiry about abuse and a crucial link to further support from the specialist DVA sector. This engagement has a strong evidence base, particularly the landmark IRIS (Identification and Referral to Improve Safety) trial [26] and subsequent interrupted time series [27], showing effective implementation and cost-effectiveness [28]. The IRIS programme, which is facilitated and monitored by IRISi, a social enterprise [29], has been commissioned in 48 areas and has trained over 1,000 practices (> 12%) in England, Wales, the Channel Islands and Northern Ireland with over 20,500 female patients referred from these practices in the past 10 years.

The implementation of lockdowns in the UK resulted in the majority of medical consultations in primary care shifting to audio or video encounters with patients [30, 31]. Lockdown-specific barriers to disclosure of abuse [32] and barriers to general practice consultations linked to the system switch to remote consulting [33] have occurred at the same time as the incidence of abuse has increased [34]. The overall drop in consultations [30] and diagnoses [35] during the pandemic has, however, reduced the opportunity for detection. DVA also remained easily hidden by the many varied presentations around common mental health problems linked to the pandemic [36, 37].

The necessary transition to remote clinical consultations has uncovered safety and confidentiality concerns, practical challenges and knowledge gaps about how general practice clinicians can ask about abuse, respond appropriately and provide ongoing support [38]. The requirement for additional skills and competencies for the management of telemedicine has also emerged. IRISi has developed and disseminated guidance [39] for general practices nationally on conducting safe remote consultations both directly and via the Royal College of General Practitioners (RCGP), National Health Service England (NHSE), and social media. The guidance raised awareness about the heightened risks and provided advice on overcoming barriers to enquiry, identification and referral to specialist services. Local IRIS providers are offering COVID-19-adapted online training to support implementation of the guidance and DVA general practice work in general.

### About PRECODE

#### Aims and objectives

In this paper we summarise the protocol for PRECODE (PRimary care rEsponse to domestic violence and abuse in the COvid-19 panDEmic: interrupted time series and qualitative study) which aims to understand the impact of the UK national COVID-19 lockdowns on general practice responses to DVA. Using a rapid mixed method design, the study will explore the impact of the pandemic on the number of patient referrals from IRIS trained general practices to DVA agencies in the UK. It also aims to understand the experiences of primary care clinicians responding to patients affected by DVA during the pandemic and adaptations they have made transitioning to online DVA training and to remote DVA identification, support, and patient referral.

#### 1.3.2. Research questions

To achieve these aims, the study will answer the following research questions:

1. What is the impact of the national COVID-19 lockdowns in the UK on the referral rates of patients affected by DVA in practices that have had IRIS DVA training and a specialist referral pathway?
2. How, if at all, have these practices adapted to online DVA training and to remote DVA identification, support, and patient referral?

The integration of quantitative and qualitative approaches (workstreams) will enable the articulation of multiscale trends in primary care responses to DVA and complex mechanisms by which these responses to patients have changed during the pandemic.

#### Patient and public involvement and engagement

An established patient and public involvement and engagement (PPI&E) group of women survivors of DVA (supporting the REPROVIDE research programme [40] and meeting quarterly) has advised on the development of the proposal and will be consulted regularly throughout the duration of the study. We have also formed a dedicated PRECODE PPI&E group of survivors consisting of women who have experienced DVA and who have been supported by a DVA specialist service and local IRIS programmes. The PRECODE PPI&E group will meet quarterly and will work in close collaboration with the research team advising on study design, conduct, analysis, interpretation of findings and dissemination. In addition, we will also consult an established male DVA survivor group that advises on the REPROVIDE programme. All our PPI&E group members will have careful induction to ensure appropriate conduct, the safety and wellbeing of the group, and to enhance their engagement. The PPI&E meetings take place in a supportive and safe environment and are followed by de-briefing sessions. Members will be supported by their DVA organisations and will be also encouraged to seek additional support from our partner DVA agencies and from Bristol Medical School's Centre for Academic Primary Care PPI&E facilitator [41].

#### Ethics and funding

The study received HRA (Health Research Authority) and Health and Care Research Wales (HCRW) Approval (20/HRA/5873) and University of Bristol Faculty of Health Science Research Ethics Approval (113,044).

In addition, the University of Oxford, Medical Sciences Inter-Divisional Research Ethics Committee (IDREC) granted amendment approval to our aligned study exploring the experiences of general practitioners with remote consultations in non-IRIS practices in the UK ‘R69839/RE003—Understanding GP (general practitioner) perspectives on the safe and effective delivery of safeguarding’ to conduct a secondary analysis and integrate findings with the PRECODE study.

PRECODE is a 12-month third sector-cross universities collaborative project and is funded by the UKRI (UK Research and Innovation) Rapid Response Call and the (MRC) Medical Research Council MR/V041533/1.

## Methods and design

### Quantitative workstream: impact of COVID-19 social distancing measures (lockdowns) on referral rates of patients affected by DVA

#### Aims and objectives

We will analyse the numbers of referrals from general practices to the IRIS service to:

(i) quantify the impact of the three national COVID-19 lockdowns and social distancing restrictions on referrals to IRIS services during 2020 and 2021
(ii) quantify the difference in the number of referrals during school holidays (periods when the number of IRIS referrals consistently dip) over different years between 2017–2020 and contrast these to referrals during the national lockdowns in 2020 and 2021
(iii) determine the key factors significantly associated with increased referrals during school holidays and during the national lockdowns in the period between 2017–2021.

#### Measurements and data collection

We will quantify the number of general practice referrals of female patients to a local IRIS programme for specialist DVA support from March 2017 to September 2021. We will use routinely collected practice-level referral data from 33 sites where IRIS was commissioned for at least 12 months before the UK announced its first national lockdown on the 23^rd^ of March 2020. In line with our previously published work [42, 43] data will include anonymised daily individual-level data for referrals to the IRIS programme received by DVA specialist agencies from general practices across 33 sites between 24/03/2017- 22/9/2021 for female patients aged 16 and above, registered at a general practice.

It is estimated that 14,000 to 15,000 individual level referrals will constitute the final sample size, given that between 1^st^ April 2017 and 1^st^ April 2020 IRIS has received 4,934 referrals to the programme.

#### Statistical analysis

Two separate but related analyses will be completed as part of this workstream.

In the first analysis, we will evaluate the impact of the pandemic and associated lockdowns on referrals to the IRIS Programme (referrals to IRIS advocate educators in specialist DVA services) and compare these to the referrals during school holidays between 2017–2020. We will undertake interrupted-time series (ITS) [44] and non-linear regression analysis, including sensitivity analyses, on time series of referrals to DVA services from routinely collected practice level data across 33 local authority or Clinical Commissioning Group (CCG) areas. We will fit different regression models to the data. For each regression model, we will calculate the Akaike Information Criterion (AIC) and the Bayesian Information Criterion (BIC) to compare models and choose the best-fit model based on the smallest values of these quantities.

For the best-fit model, we will report incidence rate ratios (IRR) and 95% confidence intervals as indicators of change in referrals before and during the first, second and third national lockdowns in 2020 and 2021 and during the school holidays in 2017/2018 and 2018/2019. This will allow us to quantify and contrast the impact and significance of the lockdowns and school holidays on the referrals. The periods of analysis will be: 01/4/2017–31/12/2019 (initial analysis of the impact of school holidays); 24/3/2019–23/9/2020 (initial analysis of the impact of the first national lockdown), 24/3/2019–22/9/2021 (final analysis of the impact of all three national COVID-19 lockdowns). The final analysis will also allow us to track the effect of reopening society following the third national lockdown in 2021 after the roll-out of the COVID-19 vaccination campaign.

The second analysis will explore key determinants associated with increased referral to the IRIS programme. We will explore whether these were different in the period before the pandemic (April 2017-December 2019) and during the pandemic (January 2020-December 2020). We will also explore the impact of training and guidance on referrals, given that within two weeks of the implementation of national social distancing/isolation policy and the shift to remote consultation, all IRIS practices were issued guidance and were offered COVID-19-specific online training to support implementation of the guidance on safe remote consultations. We will have area-level data on number of trainings that took place online. In a difference-in-difference analysis of referrals data, we will estimate the effect of that training. If possible, we will run an additional sensitivity analysis, using the local prevalence of DVA as reported to the police, based on the local Joint Strategic Needs Assessment (JSNA) or local office for policing and crime to adjust the ITS model in relation to incidence, once a chance in incidence may cofound that analysis of referral rates. Regression results for this second analysis will be presented as marginal effects or incidence rate ratios.

All quantitative analysis will be carried out using Stata MP 15.

### Qualitative workstream: understanding adaptations to remote domestic abuse consultations, referral and training

#### Aims and objectives

We will conduct an interview- and observation-based qualitative study to understand:

(i) the feasibility and utility of online training, the extent to which clinicians were able to implement national guidance and online training on safe remote consultations, support, and patient referral
(ii) primary care clinicians’ views and experiences responding to affected patients during the pandemic and offer DVA referrals to specialist services
(iii) organisational and individual practice adaptations and strategies addressing concerns and mitigating challenges around consultation safety, confidentiality and efficacy as a result of transitioning to telemedicine.

#### Sampling and recruitment

We will conduct interviews with professionals who are key in either providing support to patients affected by DVA or in reconfiguring how practices implement remote consultations. Participants will include (1) practice managers and practice administrative staff who managed or facilitated the move to remote consulting; (2) primary care clinicians who conducted routine and urgent primary care clinical consultations during the pandemic; (3) IRISi regional managers who oversaw and supported the implementation of the IRIS programme; and (4) IRIS advocate educators who (i) supported and provided on-going training for general practice teams to help them understand and respond to DVA during the pandemic (ii) received and responded to domestic abuse referrals for patients affected and provided specialist support to them.

Participants will be identified using purposive maximum variation sampling of professionals from up to 15 IRIS trained general practices, interviewing approximately 30 primary care practice clinicians and 15 non-clinical professionals (regional managers, advocate educators and practice managers). We will apply a multi-stage sampling framework. We will select up to eight sites where the IRIS programme is commissioned, usually a CCG or local authority area, during the first stage of sampling (area selection). The area selection will be informed by the initial analysis of referral rates/patterns from the quantitative workstream examining referrals across different regions and variations in DVA referrals before and during the first UK pandemic wave. Area selection will be also informed by patient population size and demographic and socio-economic composition. Sampling decisions will be guided by insights from IRISi regional managers either through formal interviews (see above) or via co-production with IRISi.

The second stage of sampling will take place within selected geographic areas and will be based on general practice referral rates. In partnership with local advocate educators, who hold links with general practices and have local knowledge of the implementation of the IRIS programme in general practices, we will identify two dissimilar general practices (for example historically high and low referrers; uncharacteristically higher or lower referrers during the pandemic). Finally, our sampling approach, using a ‘snowballing’ sampling technique for the identification of individuals, will also consider the inclusion of participants from diverse demographic groups, those with diverse professional backgrounds and experiences, and those with different levels of involvement in the IRIS programme.

We will exclude professionals from the qualitative study who were not actively in post for at least three months from the beginning of the first national lockdown (23/03/2020) (i.e. did not conduct patient consultations (primary care clinicians) or did not deliver at least one online training session (advocate educators)).

Local advocate educators will be asked to share details of the study (study summary and invitation letter) with the practice manager and a known primary care clinician with experience in IRIS referral from each practice. The clinician will be asked to recommend another clinician colleague from their practice with less involvement with IRIS to join the study. Participants will be asked to directly contact the researchers who will explain the study and will share the information sheet. If they indicate to the researcher that they are willing to take part in an interview and meet the sampling and study inclusion criteria, a suitable interview time will be arranged with the individuals directly or via the practice manager. Up to eight local advocate educators and regional managers working with different sites and regions will also be invited to participate in an interview. We will seek consent from individuals to participate in the study and to being audio-recorded prior to the commencement of the interview.

We will also observe ten remotely delivered IRIS training sessions. Our sampling units for the observation will be general practices, not individual clinicians. Therefore, clinicians for the training observation will be recruited as a clinical team via the practice manager. Observed practices will be sampled to represent a mixture of practices by geographical location, demographic and socio-economic composition of population, practice size and IRIS referral rates. Practice recruitment will be supported by IRISi regional managers and guided by local advocate educators. We will seek permission to observe training sessions from advocate educators, practice managers and clinicians.

#### Data collection

Semi-structured interviews will be conducted with general practice clinicians, advocate educators, regional managers, and practice managers remotely (via phone or video call). The interviews will be focusing on experiences of the management of DVA in primary care during the pandemic and views about the relevance, feasibility and utility of online DVA training. The semi-structured interviews will explore concerns with and experiences of asking (or not) about domestic abuse; relevance and availability of guidance; obstacles to and strategies for offering support and referral. Interviews with practice managers, regional managers and advocate educators will give insights into local adaptations and organisational and practical barriers and facilitators to delivering online training. Interviews will be audio recorded and transcribed verbatim.

We will also remotely (via video call) observe approximately ten online training sessions. The observations will document the context and dynamics of training sessions, variations in training delivery, participants’ engagement with and reflections on the content and participants’ questions and concerns. Observations will be guided by an observation framework and will be summarised in detailed field notes.

#### Analysis plan

We will apply rapid qualitative analysis techniques adapted from McNall and Foster-Fishman [45] and Vindrola-Padros et al. [46]. After each interview/observation the researcher will prepare a Rapid Assessment Procedure (RAP) summary sheet [47] to provide familiarisation and research team access to the qualitative data. These short summaries will be shared among the research team in advance of full transcripts and fieldnotes becoming available, enabling discussion of developing themes and iterative adaptation to topic guides. The qualitative research team will meet regularly to carry out group analysis of full transcripts and observation fieldnotes and to develop an initial coding framework following the Framework Method [48] as appropriate for rapid group analysis. Once the framework is agreed it will be applied across all transcripts and fieldnotes and data will be charted in a shared excel spreadsheet. At this stage, researchers will explore different framework categories for thematic insights, and will conduct further sub-analyses as deemed relevant for answering the initial research questions, as well as responding to unanticipated topics within the data.

### Synthesis of quantitative and qualitative workstreams

We will synthesise key initial findings from the qualitative and quantitative workstreams. The synthesis will lead to new insights beyond those identified in each workstreams. Exploratory and explanatory sequential mixed method data analysis [49, 50] will involve using the initial findings of the ITS to sample areas for interviews, based on variation in referral rates. Further ITS analysis will proceed in parallel with the qualitative interviews, leading to the synthesis stage in which we will triangulate the findings of the complete ITS with qualitative findings. 

We will explore temporal variation in the referral rates and variation between areas with interview data, generating hypotheses [51] to explain the variations. The initial findings of the ITS are likely to show a reduction in IRIS referrals from general practice, despite other external evidence (from national DVA helpline, domestic homicide data, police reports) that incidence rose during the first and subsequent lockdowns. The interviews with primary care clinicians will explore possible explanations for reduced referrals including reduced access to appointments and consultations, barriers to disclosure and perceptions of what DVA agencies can offer patients who do disclose. We will use timelining [52] to map clinician experiences of asking about and referring (or not referring) patients affected by DVA. The timeline will correspond to the time window of the ITS analysis, but extended to the date of the interview, to capture changes in their consultation and referral behaviour and external circumstances. Interviews with advocate educators, regional managers and practice managers on their experience of organising and delivering DVA support during the pandemic will help contextualise the ITS and clinician interview data while also comparing and contrasting primary care clinician referrals with patient self-referrals to DVA agencies. The quantitative and qualitative workstreams will be given equal significance.

We will contextualise the findings in the light of learning from parallel rapid research that has been undertaken during the pandemic and emergent policy and practice outputs relevant to the study. In particular, we will synthetise our findings with an aligned study on the experiences of non-IRIS general practitioners providing DVA support during the pandemic [53]. Our PPI&E groups’ insights regarding experiences of DVA survivors seeking support from primary health care and domestic violence services remotely will be informing the synthesis and their commentary on our findings will be integrated into the analysis.

## Discussion

### Relevance

The PRECODE study will combine quantitative and qualitative methods to explore and explain the dynamics of DVA referrals before and during the COVID-19 epidemic in the UK. We will be focusing on new evidence gaps created by the uncertainty about how general practices responded to (live) online DVA training and how they adapted their consultation methods in relation to DVA in the context of the COVID-19 pandemic. The importance of filling these gaps is driven by the prevalence of DVA globally and the UK [5, 13, 14, 34] with numbers likely to increase in the coming year(s), requiring an effective response from the NHS, particularly general practice.

The imposing of severe lockdown measures during the COVID-19 pandemic led to closure of face-to-face DVA services and consequential reduction in DVA referrals in the UK (despite increased DVA prevalence). Evidence-based, continual and effective remote support of DVA survivors and referrals to DVA service providers is crucial, as it is likely that even when social distancing is relaxed, a smaller proportion of general practice consultations will be face-to-face [54]. Analysing the change in referrals and the adaptation of clinical responses to DVA during the pandemic is crucial in underpinning future planning, and this is the first mixed-method study to do this on a national scale using qualitative interviews and over four years of referrals time series data.

### Strengths and limitations

Key strengths of our study design are the rapid mixed method synthesis of diverse evidence sources. Both the quantitative and qualitative methodologies of the study are well-established and widely used technical frameworks. The mixed method approach will allow for a broader and more granular exploration of the primary care response to DVA during the pandemic than quantitative or qualitative methods alone. The triangulation of quantitative and qualitative findings using rapid analysis and synthesis methods successfully applied previously in the context of COVID-19 research [55, 56], will allow for a rapid assessment of the variation, relevance, feasibility, and safety of primary care responses to DVA before and during the pandemic and its aftermath.

Further strength lies in the multi-professional/multi-agency collaborative approach linking general practice to the DVA sector. The study team members have led the field of domestic violence and health research in the UK for almost two decades, combining quantitative (epidemiological studies, trials, surveys, economic analyses) with qualitative methods (cross-sectional and longitudinal interview and ethnographic studies) and systematic co-production of research with third sector partners. Team members have diverse expertise around the development, delivery and testing of DVA interventions. Our landmark IRIS trial is the basis of a nationally commissioned training and advocacy programme linking general practice to the DVA sector. Team members work to improve and promote the healthcare response to DVA in the UK and internationally, work in strong partnership with service users, the DVA sector, including close collaboration with IRISi, and the RCGP, and they seek opportunities to disseminate findings internationally and to influence UK policy. Team members also lead evidence-based innovation in the health care response to DVA and are committed to influencing system change within health services and the DVA sector.

The study team will actively involve three service user expert groups. Throughout they will provide valuable insights into the perspectives and experiences of survivors. Finally, the study will benefit from including the views of different professional groups with expertise and experience in DVA and without a specific role in this area.

There are three possible limitations to this study. The first limitation concerns the possible generalisability and transferability of the findings to non-IRIS practices. Although we will ensure the diversity of recruited practices in terms of size, location and population, as well as the diversity of research participants, as the study is focusing on the pandemic responses of IRIS-trained general practices to affected patients, the findings will not necessarily be applicable or relevant to non-IRIS trained practices (consisting the majority of general practices in the UK). We will mitigate this by integrating our work with parallel research exploring the experiences of general practitioners with remote consultations in non-IRIS practices in the UK [53]. This triangulation work will support the development of resources and guidance to all clinicians working in primary care, with or without IRIS specific training. These resources will be developed with input from primary health care stakeholders from both IRIS and non-IRIS practices to ensure their applicability and relevance.

The second limitation is potential participation bias: the views of general practice professionals participating in the interview study might reflect the narratives of those individuals who may have been more experienced in or more engaged with the management of DVA identification and referral or more favourably disposed to the IRIS programme. Equally, advocate educators participating in the interview study might reflect the views of a self-selected group of professionals experienced in delivering DVA training and support.

Finally, the lack of patient research participant voice within the study will limit the interpretation of findings. As a result, although the perspectives of professionals will give indication of some of the barriers that might prevent patients from disclosing DVA in general practice, and the study will be guided by the perspectives of PPI&E members, our findings will not fully explain why some people affected by DVA do not seek or accept professional support during the pandemic. We will endeavour to interpret our findings in the light of relevant academic and policy outputs exploring survivor experiences of the pandemic.

### Implications for practice

Enabling services to respond effectively to DVA is a UK policy priority [23, 57–59]. Our study will support the implementation of this policy by generating and disseminating mixed-method evidence about the primary care response to DVA during the COVID-19 pandemic. Our findings will inform primary care and DVA service responses in the UK and other countries implementing remote clinical consultations, as they re-configure during the pandemic and beyond, including the interface between DVA and the delivery of primary care, training, and support for patients identified by front-line practitioners. We will formulate specific recommendations to improve online training and guidance on how primary care clinicians can safely and effectively address DVA in remote consultations. The recommendations will consider the needs of both IRIS and non-IRIS trained practices and ways in which DVA training and resources can be relevant and more widely available to general practices across the UK.

Given the highly sensitive nature of DVA research, we will adopt trauma-informed dissemination and data sharing approaches, still consistent with open science [60, 61]. Findings from our synthesis will directly inform policy on training and support for general practices by establishing bi-directional communication with policy makers, commissioners, health service providers, service users, and third sector organisations. As co-produced research with IRISi, the RCGP and PPI&E members, we will rapidly funnel evidence to support policy and practice nationally. Our evidence, resources and guidance will be open access and available to policymakers, commissioners, services, and front-line practitioners. We also expect to inform future national calls for evidence that feed into policy about DVA, local needs assessments and commissioning of both health and frontline DVA programmes and services.

As with other health inequalities and adversities during the pandemic, although DVA has been made more visible in the last year, the health and domestic abuse sectors are struggling globally to re-configure services and develop new strategies. The study will drive inter-sectoral UK policy with relevance globally, by contributing resources and guidance for primary care clinicians addressing DVA using remote consultations. These will be vital in supporting safe and effective care for affected patients as primary care re-formats post-pandemic.

## Data Availability

Given the highly sensitive nature of DVA research, we will adopt trauma-informed data sharing approaches, still consistent with open science. Anonymised transcript (if safe and appropriate) will be stored on the University of Bristol’s Research Data Service Facility. *Bona fide* researchers will be able to access non-identifiable data upon reasonable request. Access will be subject to a data access agreement and following approval from the Chief Investigator and the University of Bristol Data Access Committee.

## Abbreviations

AIC: Akaike Information Criterion
BIC: Bayesian Information Criterion
CCG: Clinical Commissioning Group
DVA: Domestic violence and abuse
GP: General practitioner
HCRW: Health and Care Research Wales
HRA: Health Research Authority
IDREC: Inter-Divisional Research Ethics Committee
IRR: Incidence rate ratios
IRIS: Identification and Referral to Improve Safety
ITS: Interrupted-time series
JSNA: Joint Strategic Needs Assessment
MRC: Medical Research Council
NHSE: National Health Service England
NICE: National Institute for Health and Care Excellence
ONS: Office for National Statistics
PPI&E: Patient and public involvement and engagement
PRECODE: PRimary care rEsponse to domestic violence and abuse in the COvid-19 pandeDEmic: interrupted time series and qualitative study
RAP: Rapid Assessment Process
RCGP: Royal College of General Practitioners
UK: United Kingdom
UKRI: UK Research and Innovation
